# From reflection diaries to practical guidance for transdisciplinary research: learnings from a Kenyan air pollution project

**DOI:** 10.1007/s11625-023-01317-0

**Published:** 2023-04-19

**Authors:** Heather D. Price, Cressida J. Bowyer, Patrick Büker, Cindy M. Gray, Matthew Hahn, Fiona Lambe, Miranda Loh, Alexander J. Medcalf, Timothy Kamau Njoora, Charlotte Waelde, Megan Wainwright, Sarah E. West

**Affiliations:** 1 Biological and Environmental Sciences, University of Stirling, Stirling, UK; 2 Faculty of Creative and Cultural Industries, University of Portsmouth, Portsmouth, UK; 3 Deutsche Gesellschaft für Internationale Zusammenarbeit (GIZ) GmbH, Bonn, Germany; 4 School of Social and Political Sciences, University of Glasgow, Glasgow, UK; 5 Theatre for Development Practitioner, Folkestone, UK; 6 Stockholm Environment Institute, Stockholm, Sweden; 7 Institute of Occupational Medicine, Edinburgh, UK; 8 Department of History, University of York, York, UK; 9 Department of Music and Dance, Kenyatta University, Nairobi, Kenya; 10 Centre for Dance Research, Coventry University, Coventry, UK; 11 Department of Anthropology, Durham University, Durham, UK; 12 Stockholm Environment Institute - York, Department of Environment and Geography, University of York, York, UK

**Keywords:** Transdisciplinarity, North–South partnership, Reflexivity, Reflection diary, Air pollution, Participation

## Abstract

Transdisciplinary research (TDR) approaches have been cited as essential for overcoming the intractable sustainability challenges that the world is currently facing, including air pollution, water management and climate change. However, such approaches can be difficult to undertake in practice and can consequently fail to add value. Therefore, examples of what works in practice (and what does not) are helpful to guide future research. In this study, we used a conceptual TDR framework as the basis to examine and evaluate the strengths and weaknesses of our approach in a project exploring air pollution in an informal settlement in Nairobi, Kenya. Reflection diaries exploring experiences of participation in the project were undertaken by the project team (comprising academic and community partners) at multiple time points throughout the project. These reflection diaries played an important role in evaluation and for providing space for team learning. Diaries were thematically coded according to the TDR framework to explore aspects of the project that worked well, and areas which presented challenges. We draw upon our reflections, and the extant literature, to make practical recommendations for researchers undertaking TDR projects in future. Recommendations focus on three key project stages (pre-funding, funded period, post-funding) and include; building the team in a way that includes all key stakeholders in relevant and appropriate roles, giving everyone sufficient time to work on the project, and ensuring regular and open communication. Building these recommendations into the design and delivery of transdisciplinary sustainability science projects will support progress towards achieving the Sustainable Development Goals (SDGs).

**Supplementary Information:**

The online version contains supplementary material available at 10.1007/s11625-023-01317-0.

## Introduction

The world is facing numerous simultaneous and multidimensional socio-environmental crises. Targeted by the 2030 Sustainable Development Goals (SDGs), these include waste management, climate change, food security (e.g. Steffan et al. 2015), and not least air pollution. Such complex and hard-to-solve issues often require the kind of interdisciplinary research approaches found within sustainability science (Stephenson et al. 2010). Environmental issues are “inseparable from the tangled skein of human perceptions” (Little 2017, p. 4), and therefore academic researchers from the arts, culture, humanities, natural and social sciences are required to understand interactions between humans and the environment (Heras et al. 2021), and to help develop solutions and promote change. However, sustainability science has often struggled to move from describing problems to providing actionable solutions (Wiek et al. 2015). Transdisciplinary research (TDR) moves beyond crossing disciplines (as in interdisciplinary work) to integrate different types of knowledge, such as community knowledge, throughout the research process to tackle problems from a more holistic perspective (Lang et al. 2012). This links to the SDGs, where the importance of bringing multiple partners together, including civil society organisations and individuals, is highlighted in Goal 17 (Partnership for the Goals). Applying such TDR approaches to sustainability science has the potential to move from describing the sustainability problems we face to developing and implementing shared potential solutions that are both scientifically robust and socially relevant.

It is increasingly acknowledged that the iterative and emergent nature of TDR approaches are particularly useful for addressing challenges in urban contexts in Africa (see for example Ambole et al. 2019; Buyana et al. 2019; Mulligan et al. 2020; Thondhlana et al. 2021), where weak governance systems and high levels of inequality, corruption and informality are common features (Patel et al. 2022; van Breda and Swilling 2019; Thiam et al. 2021). In her review of a set of African TDR case studies, Patel points to the important role that TDR approaches play in post-colonial societies, both in tailoring interventions to meet situated, local needs, but also in “shifting the political economy of research on Africa” by centering the research contributions of African academics (Patel et al. 2022, p. 12). In their review of the same programme, Odume et al. point to the challenges encountered in the “joint conceptual threshold crossing” that is required for true knowledge co-production to take place, and to the need for institutional linking (Odume et al. 2021, p. 121). Reviewing another suite of TDR projects funded in the SDG space in Africa, Thiam et al. (2021) also found institutions working independently, and highlighted the importance of workshops where data sharing and collaboration helped to break down the institutional silos. In such workshops and other spaces provided by TDR projects, transformative social learning can take place to support responses to sustainability challenges such as energy crises.

Lang et al. (2012) note that TDR needs to meet three requirements. It must (a) focus on societally relevant problems, (b) enable mutual learning by academic researchers and actors outside academia, and (c) create solution-oriented knowledge which is transferable to practice. In Lang et al. the researchers compiled a list of TDR design principles based on the literature and their own experiences, and gave examples of how they can be achieved. These twelve design principles fall into three different project phases: collaborative problem framing and building a collaborative research team; co-creation of solution-oriented and transferable knowledge through collaborative research; and (re-)integrating and applying the co-created knowledge. This three-phase framework has come to typify much TDR, with versions of the framing applied in theoretical as well as empirical studies (e.g. Brandt et al. 2013) and used as a tool for monitoring, evaluation and learning during project implementation (Siew and Döll 2012, Siew et al. 2016).

Although the benefits of transdisciplinary working are widely acknowledged, undertaking TDR can prove to be challenging in practice. At a fundamental level, implementing such projects is time consuming due to the requirement for ongoing, adaptive and intensive engagement with stakeholders (Hoffman et al. 2019; Thomson et al. 2017). There can also be difficulties managing differing expectations amongst team members (Polk 2015). Furthermore, issues can arise relating to power dynamics and local politics (Marshall et al. 2018), particularly if insufficient time is given to build trusting relationships (Stauffacher et al. 2008). This is a problem that has been exacerbated since the COVID-19 pandemic began, due to much reduced international travel and a lack of in-person meetings, thereby hampering trust building processes and the implementation of fieldwork (Hall et al. 2021). Whilst digital and online approaches have proven appropriate and efficient for project meetings and administrative tasks, fieldwork, stakeholder engagement, brainstorming and spontaneous and creative interactions between participants are less successful in the digital space (Smidvik et al. 2020). Some TDR projects fail to deliver new scientific insights, with project benefits being realised predominantly for non-academic stakeholders (Lang et al. 2017). For other projects, a lack of robust synthesis and impact evaluation means the potential for the project to deliver real change is not realised or remains undocumented (Hoffman et al. 2019; Huang and Harvey 2021). Other, longer term impacts can be difficult to capture, or take place after the project has finished.

Evaluation is a key component of TDR projects (Bergmann et al. 2005; Lang et al. 2012; Binder et al. 2015), but can be very challenging to undertake (Hellström 2015). Formative evaluation, that which is conducted whilst the project is underway in order for the team to learn, is particularly important for TDR projects, however, there is a lack of understanding of how best to do this in practice (Ding et al. 2020). Team reflection is a process of recognising and exploring issues which can result in learning, and therefore can play a useful role in formative evaluation. This learning is vital for helping ensure TDR projects maximise their potential utility for solving socio-environmental crises. Reflective diaries have commonly been used, particularly in education and medical practice, to support individual reflection and self-learning (Meth 2003; Cohen et al. 2006); however, their use in team reflection has been much less common. If undertaken successfully, reflexivity in TDR projects has also been highlighted as one way of recognising and addressing power imbalances within TDR projects (Steger et al. 2021).

Air pollution is an example of an intractable, global sustainability challenge that can benefit from TDR approaches (Ebi et al. 2020). The World Health Organisation (WHO) estimates that 9 out of 10 people breathe air that contains high levels of pollutants (WHO 2018). Africa is particularly badly affected by air pollution, where fine particulate matter (PM2.5) accounts for an estimated 920,000 premature deaths each year (Forouzanfar et al. 2016). Children, the elderly and those with cardio-vascular diseases are particularly vulnerable to the effects of air pollution. Academic researchers from the natural science disciplines are studying the issue and developing technologies to mitigate negative effects, but there has been insufficient collaboration between the natural sciences and the humanities and social sciences to explore how air pollution is perceived in cultural terms, and therefore how society as a whole might more effectively address it (Little 2017).

This article critically examines a TDR network-building project focused on air pollution in Nairobi, Kenya, identifying and discussing elements of the project design and implementation that worked well and those that were not so successful, using reflective diaries and the highly cited Lang et al. (2012) design principles as a framework. Although various guidelines and conceptual frameworks have been developed for the evaluation of TDR projects (see for example, Brink and Wamsler 2018; Pohl and Hirsch Hadorn 2007; Schneider and Buser 2018), we selected the Lang framework since it “provides a targeted, specialist overview, specifically from the sustainability science perspective” (Lawrence et al. 2022, p. 45). We use our reflections on this project, and literature around TDR practice, to make practical recommendations for people designing and undertaking TDR projects, including using reflective diaries as a means of documenting projects and facilitating deeper learning about successes and failures.

The article is organised as follows: section two provides an overview of the case study and describes the methods applied and how the reflective diaries were analysed; section three presents and discusses the results; section four provides our recommendations for TDR project implementers and funders; and section five outlines our conclusions. Taken together, these underscore a number of core recommendations around representation, flexibility at all levels, and opportunities to challenge.

## Methods

### Project summary

In this study, we analysed the strengths and weaknesses of our approach in undertaking a TDR project on air pollution in Kenya. The aim of the project was to build a research network of Kenyan and European academic researchers and Kenyan community partners, with the long-term purpose of creating innovative, participatory solutions to air pollution and its effects on human health in low-resource settings in Sub-Saharan Africa. We used a community-based participatory approach in Mukuru, an informal settlement in Nairobi, Kenya. Financial support was provided by a UK funding agency (MRC/AHRC) under Global Challenges Research Fund for 18 months (October 2017–March 2019) and the project was led by a UK-based research organisation. The project comprised 17 academic researchers from ten partner organisations in the UK, one in Sweden and four in Kenya. The team was purposefully diverse in terms of the disciplines and backgrounds represented. Although we recognise that disciplines themselves evolve and have fuzzy and sometimes contested boundaries (Vick 2004), the team was drawn from academics working in anthropology, law, creative arts, chemistry, environmental sciences, storytelling, history and geography, while 19 community partners came from professions including teachers, health workers, visual artists, and musicians. Community partners were paid a day rate, as recommended by Kenyan partners, for their involvement in the project.

**Fig. 1:**
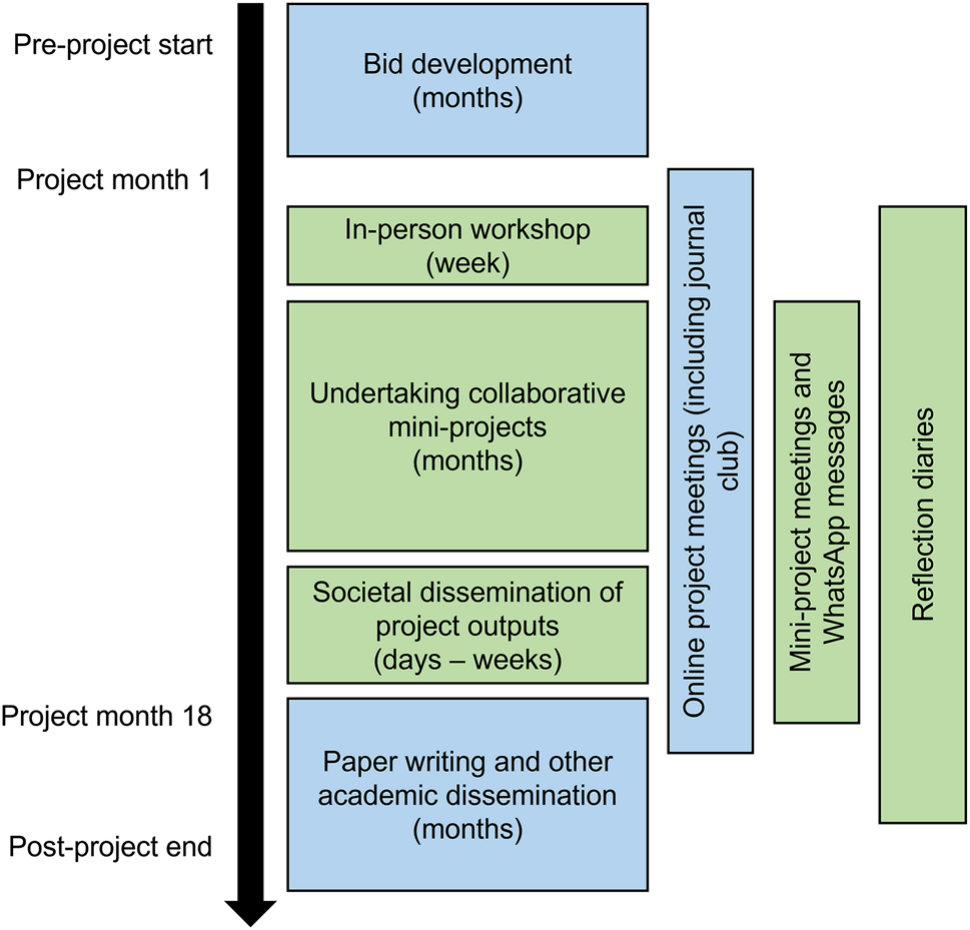
Summarised project timeline highlighting key activities and the people involved. Blue boxes show that the activity was undertaken by academic researchers only and green boxes show that the activity was undertaken by academic and community partners collaboratively. Indicative timings for particular activities are given in brackets

A summary of the key project activities (and associated timelines) is given in Fig. 1. These activities began with a 1 week in-person workshop held in Mukuru, Nairobi, Kenya. At this workshop, which included both academic and community partners, much of the collaborative problem framing and building of a collaborative research team (Phase A; Lang et al. 2012) and co-creation of solution-oriented and transferable knowledge through collaborative research (Phase B; Lang et al. 2012) took place. This included the co-design of ‘mini-projects’ (budgeted for within the main project funding as ‘flexible funds’ dependent on the co-design process), within which the bulk of the research activities took place. Academic and societal outputs were various, with societal outputs generated before academic outputs (Phase C; Lang et al. 2012). Throughout the project we used various mechanisms to support participation, engagement and communication including online project meetings, WhatsApp messaging and reflection diaries. For further details of the project discussed, see West et al. (2021).

### Data collection: reflection diaries

Reflective practice has become an important part of professional education over the past few decades (Boud 2009), but is not commonplace in many research settings. An exception is health research, where individual solicited diaries are commonly used (e.g. Meth 2003; Cohen et al. 2006). As Lang et al. (2012) highlight, continuous formative evaluation is an important part of TDR; however, Ding et al. (2020) note a gap in knowledge of how to actually undertake such team-level reflection. To help fill this gap, in this project we invited all academic and community partners to complete reflective diaries throughout the project. Here, we used them to document our experiences, as part of formative evaluation, and also so that our experiences could inform future projects. Participants were encouraged to submit a diary entry at any time, but with prompts to do so at specific points within the project. A final opportunity for reflection was given 1 year after the end of the project. Other TDR projects have reported on reflective exercises that took place after the project ended (e.g. Binder et al. 2015), but team-level formative reflection is less commonly published. Reflection diaries were completed using an online form (Google Forms) which could be completed using mobile phones. Entries were structured to aid completion, with team members invited to reflect individually on the following questions:

The thing I am particularly enjoying at the moment is…

The challenges I/we are facing at the moment are..

We are trying to work in a "transdisciplinary" way i.e. participatory knowledge production that is characterised by the inclusion of both multiple disciplines and practice-based knowledge and expertise in the knowledge production process—how well do you think we are doing this?

Something I learned from this project is….

Something I am going to apply from this project to other areas of my work is…

If I were doing the project again, I would do the following differently….

Throughout the project, at monthly team meetings, anonymised summaries of reflections were shared with the team and were used as the basis to explore potential project amendments. In some cases this led to changes being made relating to the practicalities of undertaking the project or relating to the research plans, methods and outputs (for instance, scheduling further meetings, providing extra support to help with delivery of mini-projects, or simply providing more water at the in-person workshop). As well as addressing practical issues identified throughout the course of the project, this also served to emphasise that feedback was being responded to so as to encourage future completion of reflection diaries. As is often the case with diary research, there was attrition in the number of people completing entries over time, and uneven completion (Robson and McCartan 2016). However, it proved highly useful for formative evaluation and, by reflecting on the totality of diary entries at the end of the project, allowed a deeper and more rounded evaluation than would have been possible without them.

### Data analysis

Reflection diary responses from Google Forms were stored in Google Sheets and then uploaded into NVivo (version 12). Diary entries were independently coded by two authors (HP, SW) who then compared their coding and agreed on final coding. A deductive approach to coding was applied using Lang et al.'s twelve general design principles for TDR projects as our codes:

- Build a collaborative research team;
- Create joint understanding and definition of the sustainability problem to be addressed;
- Collaboratively define the boundary/research object, research objectives as well as specific research questions, and success criteria;
- Design a methodological framework for collaborative knowledge production and integration;
- Assign and support appropriate roles for practitioners and academic researchers;
- Apply and adjust integrative research methods and transdisciplinary settings for knowledge generation and integration;
- Realise two-dimensional integration;
- Generate targeted products for both parties;
- Evaluate scientific and societal impact;
- Facilitate continuous formative evaluation;
- Mitigate conflict constellations; and
- Enhance capabilities for and interest in participation.

HP and SW then used the coded diary entries to document what worked, and did not work, at each stage of the research process, drawing on the TDR literature where relevant to draw out recommendations. Next, the other authors assisted with refining the article, drawing on their own knowledge of events to help fill in gaps where issues were not raised in the reflection diaries, and adding wider literature. All academic members of the project team were invited to read the article, make edits and add literature; those who did were added to the authorship list. Unfortunately, the majority of those who contributed to the writing of the article in one of these ways were from European backgrounds.

## Results and discussion

In total, 79 reflective diaries were completed by 17 academic researchers, with a further 25 from community partners. Academic researchers completed an average of four entries. Community partners only completed entries at the in-person workshop, mainly because most of them could not access internet facilities in their homes. This imbalance should be borne in mind when interpreting the findings and is an acknowledged limitation of the study. In the following, we present our findings according to each of Lang et al.’s design principles grouped by phase (A, B, C). The tables under each phase describe what activities and approaches were undertaken that exemplify each design principle. In the text we present researchers’ reflections on these using illustrative quotes and simultaneously frame and discuss these in relation to the work of other scholars.

Lang et al. (2012) also outline some general design principles for TDR projects, which aim to encourage an ‘effective and efficient research process for all actors involved’ (p. 35), and include facilitating continuous formative evaluation, mitigating conflict and enhancing capabilities for (and interest in) participation. For reasons of space, we have incorporated these general principles throughout the other sections.

### Phase A (collaborative problem framing and building a collaborative research team)

Lang et al. (2012) describe the first key phase in TDR projects (Phase A) as about building the collaborative research team and co-developing the team’s understanding of the sustainability problem to be addressed. In our project, activities to support our team building and collaborative problem framing included conducting an in-person start-up project workshop, co-developing a contract which outlined the ways in which we wanted to work together, holding a regular academic journal club and co-designing mini-projects with community partners (Table 1).

**Table 1:** The specific activities and initiatives undertaken within the project according to Lang et al.’s (2012) TDR design principles in Phase A (collaborative problem framing and building a collaborative research team)

Design principles (from Lang et al. 2012)	Putting the principles into practice within the project
Build a collaborative research team	The academic team was formed initially through existing contacts, with other UK partners coming from an interdisciplinary networking workshop run by the funder, and additional Kenyan partners coming via personal existing (or new) contacts of team members. The funding bid was written collaboratively by the academic team. A project advisory group was assembled, made up of UK and Kenyan academics working on health and/or air pollution. Community partners were subsequently recruited for the project start-up workshop in Mukuru by the Kenyan team Team building activities at the (professionally facilitated) start-up workshop in the community where the research was based included mapping existing connections between team members, sharing experiences, a walk around Mukuru, playing games and sharing meals, which was an important aspect of cultural sharing/bonding Academics and community partners drew-up an ‘interdisciplinary working contract’ (see ‘supplementary material’) at the start-up workshop which defined the personal attributes (e.g. openness, avoiding jargon, good listening skills) considered important for successful collaboration and how we would work together in the project Mini-projects were designed collaboratively by academics and community partners. Each was jointly led by an academic and a community partner (see below) Journal club (see below)
Create joint understanding and definition of the sustainability problem to be addressed	A monthly journal club for academics was initiated at the start of the project which spotlighted previous research from various disciplines that had links to our project Academics and community partners attended the project start-up workshop in Mukuru where the air pollution problem was jointly defined by sharing personal experiences (using stories, images or objects), listening to short scientific talks, together experiencing Mukuru’s air pollution on community walks and interactive exercises designed to prioritise the air pollution problems faced by residents
Collaboratively define the boundary/research object, research objectives as well as specific research questions, and success criteria	At the start-up workshop we defined what success meant to (1) academics (generating new knowledge to support later intervention design, linking with other ongoing air pollution projects and initiatives), (2) community partners (understanding and learning some scientific language, learning how to keep the environment clean and safe, composing a hit song) and (3) the whole team (funding to continue the research, trying something new and engaging beyond comfort zones, working in line with our ‘interdisciplinary working contract’ (see above), generating inspirational images and visuals for communication, generating better solutions to air pollution) The four mini-projects (MPs) were co-designed during the start-up workshop via a facilitated process that ensured that each MP had a mixture of skills, disciplines, academics and community partners. MP topics were identified following an interactive group task to prioritise the key air pollution issues faced by Mukuru residents: (1) raising awareness about air pollution, (2) acting against air pollution, (3) engaging with industry, and (4) prioritising policies for tackling air pollution. MP teams received iterative feedback during the workshop from the other attendees and afterwards they developed Action Plans (see below) which the project advisory group gave feedback on. Each MP had a series of objectives associated with it
Design a methodological framework for collaborative knowledge production and integration	There was close collaboration between academics and community partners during the development of the MPs as members jointly determined their objectives, the methods that would be used and outlined the expected community benefits and detailed these in Action Plans

#### Building the team

Being engaged at a relatively early stage of the project was appreciated by the community partners, for example when asked what they were enjoying about the workshop, one community partner stated: “Being engaged from the beginning” gave “a sense of being valued” (community partner 1).

The team of academic researchers and community partners that resulted from contacts, a workshop and snowball approaches was a “…varied and talented team” (academic researcher 1). However, one negative consequence of having such a broad range of academics on the project with a limited project budget was that everyone’s funded time on the project was small, at around 15 days for each academic over the course of the project. This impacted our ability to learn deeply about the history and local social and cultural contexts, which Steger et al. (2021) highlight as ‘best practice’ in TDR projects. In addition, there was an imbalance between the number of academic researchers from the UK and Kenya. This imbalance was frequently highlighted in the reflection diaries as something that could be improved by involving more academics from Kenyan universities. The imbalance in the number of European and Kenyan researchers meant that entrenched power relations between Europe and Kenya were preserved, an issue Schmidt and Neuburger (2017) discuss in their study of a North–South project involving partners from Germany, Angola, Botswana and Namibia, and is also discussed in Kareem et al. (2022).

The restricted time on the project, and the lower number of Kenyan researchers meant that we did not have well-developed links with all key local stakeholders, for example industry representatives and universities at the outset of the project. One community partner noted at the start-up workshop that to improve the project we should “Bring all stakeholders on board” (community partner 2).

However, building trusting relationships with stakeholders takes significant time (Stauffacher et al. 2008), and was ultimately unrealistic to achieve within the project’s time frame. Therefore, we relied mainly on pre-existing relationships between team members and other stakeholders and hoped to draw on these throughout the project.

#### Creating joint understanding

Building trusting relationships is particularly important when bringing together people with different backgrounds and philosophies, as people can have different understandings of what constitutes knowledge and how it is created (Stephenson et al. 2010; Ott and Kiteme 2016). Building strong relationships and trust between team members has been highlighted as an important foundation of TDR projects (Thomson et al. 2017). Regular online meetings for the academic researchers on the team helped here, as did regular WhatsApp communications among the whole team. The importance of having regular meetings, both formal and informal, was mentioned by nearly half of the publications reviewed by Ding et al. (2020) in their review of cross-disciplinary working practices. The academic journal club portion of our online team meetings enabled us to learn about different ways of thinking and was well received.

“I found the journal club…really useful…it's the kind of paper I would never normally read, but I could see how the theory underpins the research we are doing…” (academic researcher 1)

We used our start-up workshop in a community centre in Mukuru to bring together the team of academics and community partners to develop joint understanding of the air pollution issues facing the community, to decide on how we wanted to work together, and to develop plans for the small research projects [mini-projects (Table 1)]. The community partner-led walk helped develop a shared understanding of the issue. “I really enjoyed the walk and the fact that I could see what I had read” (academic researcher 3).

Workshop attendees also all participated in a programme of team building exercises, short scientific talks, storytelling and theatre, which helped to build a common sense of values and goals, and also enabled us to learn about other people’s framings of air pollution and varied ways of knowing and understanding. Steger et al. (2021) describe methods that are useful in undertaking the ‘exploration’ phases of TDR projects, where learning is being undertaken about the people involved in the project and the context in which the project will be undertaken. These include participatory action research methods (e.g. transect walks, photovoice), participatory mapping, participatory scenario planning and ethnographic methods (e.g. participant observation). Based on our study, we can specifically recommend the use of storytelling, walking, games, sharing of food and simply spending time together (Table 1). One researcher shared the impact of these methods on joint understandings: “There is so much energy in the room—it feels like there is a shared vision of what we hope to achieve” (academic researcher 1).

At the start-up workshop we also worked in groups to identify the personal characteristics of a good team member, shared these with the whole group and compiled a list which was agreed and signed by all, known as our ‘interdisciplinary working contract’. These personal characteristics, which included being patient, open-minded, flexible and respectful, mirrored those identified in previous studies (e.g. Ding et al. 2020; Steger et al. 2021). The process of making the contract was highly valued by the team. In the words of one researcher “…co-developing the contract for interdisciplinary work… really helped the process” (academic researcher 4).

Our original plan had been to develop the contract with the academic researchers only, but fortuitously the community partners arrived at the workshop earlier than expected, which allowed them to participate in this exercise, and was an important part of team-building. One of the academic researchers highlighted this when they were asked what they were enjoying in the project at the moment:

“Giving everyone the chance to input into the contract thereby giving them ownership. This process was enhanced by the fact that the [community partners] from Mukuru arrived early and took part in this process—I think this was a happy accident” (academic researcher 5).

The contract was initially intended to be used to mitigate conflict, and to encourage all researchers to abide by collaboratively outlined practices and etiquette, but in reality we did not use it to this extent.

#### Co-designing the research

The acknowledged best practices for transdisciplinary ‘science with society’ projects include collaborative design, i.e. designing together the issue to be explored, the project goals and the research questions or hypotheses (Steger et al. 2021). This was achieved by co-designing mini-projects (Table 1) at our workshop. This process was generally well received by team members; however, it was noted that contributions were not always equitable and community members should be encouraged to express themselves more:

“I feel that the process is genuinely bottom-up, democratic and inclusive” (academic researcher 6).

“In some sessions, it seemed like the discussions were between project team members [i.e. academics]. Maybe it’s because we were using “complicated” language in some sessions” (academic researcher 7).

#### Integrating knowledge

As part of the co-design process, the four mini-project teams developed Action Plans which described the aims of the mini-projects, the team (and associated roles), the methods to be used, timelines, the expected outcomes and benefits to the community. Despite the ‘criteria for success’ that were outlined for our project at the start of the workshop (Table 1), we found that the focus in the Action Plans was on the outcomes for the community rather than for science, which had consequences for the integration of the findings towards the end of the project [see "Phase C ((re-)integrating and applying the co-created knowledge)"]. This was a concern shared by several academic researchers on the team, for example:

“I'm just not sure what the research "findings" are. Perhaps this will become clear when we have synthesised our various mini projects” (academic researcher 6).

A lack of academic benefits is a risk of TDR (Little 2017). To overcome this, it has been suggested that clear objectives and methods need to be agreed at the outset (Lang et al. 2012; Little 2017), which is something we did in this project, but in our case with more emphasis placed on the community (rather than academic) benefits.

### Phase B (co-creation of solution-oriented and transferable knowledge through collaborative research)

Here we outline the mechanisms by which we sought to co-create solution-oriented and transferable knowledge. There are two key design principles for this phase as outlined by Lang et al. (2012) (Table 2). The first is ensuring that tasks and roles are clearly defined, that these are supported through resources and facilitation, and that there are low thresholds for participation. The second is around applying integrative research methods, settings or tools to generate and integrate knowledge (Lang et al. 2012). Key mechanisms we used were the journal club, the start-up workshop, mini-project Action Plans and activities.

**Table 2:** The specific activities and initiatives undertaken within the AIR Network according to the design principles for transdisciplinary sustainability projects outlined by Lang et al. (2012) in Phase B (co-creation of solution-oriented and transferable knowledge through collaborative research)

Design principles (from Lang et al. 2012)	Putting the principles into practice within the project
Assign and support appropriate roles for practitioners and researchers	For the project as a whole, academic roles were defined as part of the bid submission documentation In the mini-projects, academic and community roles were defined as part of the Action Plans (see Table 1)
Apply and adjust integrative research methods and transdisciplinary settings for knowledge generation and integration	The journal club (see Table 1) allowed exploration of different disciplines’ methodological approaches At the start-up workshop, the team tried out different methods, e.g. games, theatre, drawing, storytelling Mini-project (MP) teams were self-forming based on personal interests, around the four key issues prioritised by the team. Additional team members (with specific expertise) were brought in if necessary. The multiple methods used reflected both the collaboratively defined aim of the team, and the skills of the MP team. MP methods included photovoice, digital storytelling, participatory mapping, performative storytelling, forum and legislative theatre and policy mapping

#### Roles and responsibilities

There were two phases at which roles were defined; one was during the writing of the bid, where we had to allocate financial resources to researchers, and then the second was during the start-up workshop where the mini-projects were formed. During bid writing, we agreed that all investigators (outside of the three in the leadership team) should have the same amount of time on the project, and should attend the workshop and monthly project meetings, and engage in mini-projects. However, funding was limited and the competing demands on people’s time did cause issues with people missing meetings, as one academic researcher noted:

“There is a challenge around participation of the Co-Is in meetings and taking responsibility for taking action on things. I suppose this is a reflection of how little time we have on the project. Because I have a very flexible research job and do no teaching, I am usually able to make the meetings. So I am at or overpassing the number of hours I have paid on the project, which I'm fine with if it remains just slightly over at the end of the project. However, I wonder what will happen with colleagues who 'owe' hours to the project due to an accumulation of meetings missed. Will they make up for it in the mini-projects? In the paper writing?” (academic researcher 3).

They suggested ways of improving attendance including giving more notice, using polls to identify convenient times, and clarifying expectations for attendance. The consequences of missing meetings or parts of meetings were described in the following way by one researcher. “I had to miss most of the second meeting, and even though the notes and general communication is outstanding, it instantly creates a bit of a disconnect” (academic researcher 10).

Unfortunately, although we tried giving more warning for meetings, doing polls for dates, and making expectations clear, these did not lead to increased engagement, suggesting the fundamental issue was lack of time to engage. One of the biggest challenges when undertaking TDR, along with unequal power dynamics, is limited time (Thomson et al. 2017; Steger et al. 2021). In our project, lack of time was particularly profound after the end of the project:

“everyone is very busy with other projects etc. and so after funding ended participation tailed off for some people” (academic researcher 8).

This made article writing challenging, and although some academics were able to continue this as part of their paid employment, others were not in such a position, which was inequitable. Insufficient time is a particular problem in short-term projects like ours, which was 18 months. Ding et al. (2020) reviewed cross-disciplinary global health research and suggested that funders should provide longer term (3 years or more) funding to allow time to jointly define research problems, develop trust, and integrate knowledge.

The inflexibility of funding for TDR projects is a common challenge (e.g. Lux et al. 2019). Institutions need to be able to support inter- and transdisciplinarity, which many universities unfortunately are not, partly due to metrics (such as the UK Research Excellence Framework) being organised in subject/discipline units (Little 2017). In this project, institutional barriers included an exceedingly slow time setting-up contracts between partners and making payments to partners, leading to delays to payments for community partners:

“We have also struggled with payments—I feel like people are asking me a LOT about where the money is etc. and I'm doing all I can to chase payments, but it just isn't happening as quickly as people would like” (academic researcher 8).

The main space for discussing expectations of academic and community partners was the start-up workshop, where the interdisciplinary working contract (see Table 1) outlined ways of working, and mini-project activities were defined and action-plans developed, but unfortunately several partners did not attend the whole meeting, which caused issues later on about what the expectations of them in the project were. One academic researcher noted in the ‘Challenges’ section of the reflection diary that we needed to: “Ensure all partners have clear understanding of what is expected of them and aim of the project” (academic researcher 8)*.* Ultimately, lack of clarity on roles led to tensions and one partner pulled out six months into the project. An academic researcher had earlier noted about them: “'I’m still not super clear about what role [community partner] have in the project” (academic researcher 3). Ding et al. (2020) highlight that negative emotions are common in cross-disciplinary research, relating to unfamiliarities with this way of working. These negative emotions may have arisen due to inadequate communication about the process right at the beginning, compounded by missing meetings and not checking-in individually with them about their collaboration. An academic partner who unfortunately missed the second-half of the start-up workshop said they were unclear what their role on the project was, and at the end of the project noted:

“While there is a lot of goodwill from so many of the researchers and participants in this project, the issue of time or better yet management of time was quite a challenge for so many, at least for me personally. I do not think I have an answer yet to this situation” (academic researcher 9).

This was both an issue of lack of time and lack of clarity on roles. Although the mini-project Action Plans outlined roles and responsibilities, once mini-projects were underway, these were not always adhered to. There was a sense that the fast-paced and dynamic nature of the start-off workshop, although very positive, also got everyone a bit “carried away” making plans that were perhaps not as clearly thought through as they could have been, and which later conflicted with the actual amount of time available. This resulted in some imbalances in participation.

“You really can't expect to have a local community member co-lead the project who was nominated and agreed very quickly. We couldn't figure out in the time we had what he would need in order to participate and the result is so far there is no participation” (academic researcher 3).

“the mini projects got carried away on enthusiasm from researchers and community members, meaning that actually everyone's time was spread very thinly - the project relied on a lot of good will and over-work from many members. Some researchers, on the other hand, were not so engaged as they had other work commitments, and unfortunately this included several of the African partners so the academic side of things was, at times, very dominated by the European partners” (academic researcher 8).

Lang et al. (2012) also note the importance for scientists of balancing societal relevance with scientific rigour when it comes to roles and responsibilities. This was not very successful in our case, with more focus on societal relevance than scientific outputs, for example, through facilitating a ‘Hood2Hood’ festival, creating songs and other artistic outputs, leaving no time for scientific outputs within the period of funding.

#### Integrative research methods

The start-up workshop allowed the team to experience different ways of working and different methods, which many found stimulating and mind-expanding: “Loved the warm up exercises and [community member’s] game” (academic researcher 10). “Today researchers became actors!” (academic researcher 11). Others noted that they would take these experiences with them into future work: “I learned heaps of new methods and facilitation approaches for data collection and meetings that will for sure be useful elsewhere” (academic researcher 3).

The mini-project activities allowed close working between different disciplines, backgrounds and ways of working. As one academic researcher noted, this was valuable for many reasons:

“Working as a team, we all participated in a range of activities and exercises for knowledge production and I think it's true to say that all of us experienced something completely novel. Multiple voices were listened to, and the methods of knowledge production allowed non-experts to participate. In fact, because of the transdisciplinarity we were all both experts and non-experts at one time or another” (academic researcher 2).

TDR projects can sometimes fail to generate new scientific knowledge, because the emphasis is placed on the transdisciplinary process of working together rather than the generation of new knowledge (Lang et al. 2017). This was the case in our project, where our inclusion of many different methods on a very tight budget meant we were not able to integrate findings from the different methods. Even as early as the start-up workshop, some researchers were thinking about how all the creative methods would be integrated with science and ultimately lead to new insights.

“Great strides have been made in making the creative methodologies a central part of the project. I will wait to see how well this has worked in the sense of developing new insights as we work through outputs” (academic researcher 12).

“I feel that we are doing pretty well incorporating the creative side of things in our plans. We need to embed a bit more science” (academic researcher 2).

These concerns are not uncommon in TDR. Hoffman et al. (2019) note that the integration of research findings at the end of TDR projects (which some consider the ultimate goal) is a serious challenge. Brandt et al. (2013) describe how plurality of methods commonly used in TDR causes issues with lack of reproducibility, the cost of integrating methods, and communicating about the work. However, the advantage of being flexible in the methods used means that they can respond to the iterative development of the research project, evolving to the needs of the team (Bracken et al. 2015). In our case, the short project timeline (18 months) and primary focus of the grant being network building and experimenting with methods, left little time for integrating learning (West et al. 2021). Ding et al. (2020) highlight the need for project leaders to have explicit knowledge of integration goals, including encouraging identification of differences and discussion of these across disciplines. Pohl et al. (2021) describe integration as an interactive process that can happen throughout the course of a project. Although our monthly meetings and journal club were a potential forum for these discussions, these were dominated by reporting on the day-to-day activities of the mini-projects, leaving insufficient time for actually integrating the knowledge arising from the different activities. In our case, explicit goals around when knowledge integration was to take place would have likely helped.

### Phase C ((re-)integrating and applying the co-created knowledge)

Lang et al. (2012)’s third phase is about applying the knowledge that has been co-created through the project. There are three design principles: using the integrated knowledge to resolve or mitigate the problem, generating outputs that are suitable for both academic and community partners, and evaluating scientific and societal impact according to the success criteria agreed by the team. Table 3 shows how our activities fit into this third phase. Brandt et al. (2013) describe this phase as integration and application of results.

**Table 3:** The specific activities and initiatives undertaken within the AIR Network according to the design principles for transdisciplinary sustainability projects outlined by Lang et al. (2012) in Phase C ((Re-)integrating and applying the co-created knowledge)

Design principles (from Lang et al. 2012)	Putting the principles into practice within the project
Realize two-dimensional integration	In addition to the products (see below), the team underwent capacity-building activities, including training and experience in interviewing, theatre, storytelling, and digital storytelling
Generate targeted products for both parties	Products/outputs for society were: song and linked music video, community air pollution maps, outdoor street theatre performances and storytelling performances, legislative theatre workshop (where community members could advocate to those in power for change) air pollution murals, digital story, ‘Hood2Hood’ community festival (showcasing theatre, music, games, mapping and storytelling project outputs), radio and paper coverage (Kenyan community members and UK academic team), exhibition (York, UK) Academic products/outputs were: a journal article describing methods used and highlighting scientific findings, workshops and conference presentations including to the Kenya Air Quality Network, Defra and Medical and Health Humanities Africa, article in ‘The Conversation’, exhibition (York, UK and Gothenburg, Sweden), blog documenting project, funder website blog piece
Evaluate scientific and societal impact	At the start-up workshop we co-designed the ‘success criteria’ for academics and community partners and academics reviewed this at the end of the project We used an impact tracking spreadsheet to monitor impact activities (a requirement of UKRI funding)

#### Using the knowledge to mitigate the issue

The ‘big issue’ driving our work was the need for ways to mitigate air pollution and the harm caused to residents of Mukuru from it. However, the short-term grant’s main focus was to build a network and to some degree test-out and experiment with methods to build capacity for team members to integrate this transdisciplinary knowledge in future air pollution research and action. Therefore, the ‘smaller issue’ we were dealing with was that air pollution research, to develop solutions, had to go beyond scientific methods by integrating arts, social science and humanities approaches. The reflection diaries suggested that the collective knowledge generated was indeed inspiring team members to take forward new ideas into future work on air pollution inside and outside of academia. This capacity building happened for both academic and community partners, for example, a researcher reflected “I’m learning so much about creative/interactive disciplines—I definitely want to incorporate some of these new approaches in future projects” (academic researcher 1). Similarly, a community partner said they were learning “How to use art to tell my community about air pollution” (community partner 2)*.* Community partners also underwent formal training in interviewing, writing and producing digital stories (short videos), ethics and data handling, and learned through hands-on experiences of storytelling and theatre. Some of these community partners have since gone on to collaborate in other research projects and continued to develop skills there.

#### Generating products

The project generated a range of products for both community and academic audiences, as shown in Table 3. For more details, see West et al. (2021). Some products were temporary (e.g. theatre performances) but in other cases these were recorded and put on YouTube (e.g. Dennis’ digital story, and Mazingira song). The ‘Hood2Hood’ was an important product for the team. This was an existing format for community events, and allowed us to reach an audience, in particular youth, who would not usually hear about air pollution, as one academic researcher said:

“There is real value in injecting ideas into existing structures. In this case, relying on an established successful format of a community festival and combining it with a focus on air pollution. It opens the discussion up to a much wider range of people, although the engagement will be less in-depth” (academic researcher 10).

There was considerable community control over this event and other products generated by the project, which one academic researcher noted was a potential issue “It will be interesting to see if we manage to get 'enough' of the science reflected in the project outputs”, and one mini-project did “have unaccounted for outputs over which I have little control” (academic researcher 3), in this case a song*.* Although the song was transferred to the project, we had requested a minor edit (sound and lyric of a violent nature) and despite many attempts to contact the community partner, we lost contact and took the decision not to edit the song ourselves without explicit consent. Although the song writer and producer signed a release form at the start of the project we were not comfortable using or editing the song once they became unresponsive: in retrospect, “We should have explained again and again what the release forms meant to ensure everyone understood we would be using any materials produced and transferred to the project, even if someone withdrew from the project” (academic researcher 3).

Team member engagement waned over the course of the project. Klenk and Meeham (2017) describe TDR approaches as being a ‘double-edged’ sword, something which inspired excitement, ideas and developed new capacities, but which has many challenges around time, energy, trust and ongoing involvement. We certainly found ongoing involvement a challenge, for both academic and community partners, who understandably moved on with other things in their lives. Maintaining engagement in some of the academic-facing products that were not generated within the life of the project, in particular writing the journal article, was a challenge.

#### Evaluating impact

Evaluating societal and scientific impact of TDR projects can be challenging (Lang et al. 2012), particularly as impact usually takes place long after the end of the project. Our evaluation of the short-term impact of our project took two forms, completing an impact tracking spreadsheet for our funder which documented project outputs (including academic outputs), and defining what success would look like at our start-up workshop and then revisiting it at the end of the project. Many of our intended outcomes are what Bracken et al. (2015) describe as intangible impacts of being involved in TDR, such as increasing confidence, learning from each other, and continued engagement. Some of these we achieved, for example learning certainly took place, both about methods and ways of working, and about air pollution: “We are certainly learning things from each other”, (academic researcher 3) and “Learning more about air pollution” (community partner 2). However, other aspects, such as embedding the project within other African initiatives did not take place to a great extent, as reflected by one academic researcher:

“it seems we are missing a trick not to connect our work to ongoing research-policy processes in Kenya. However this can only work if we are invited to participate in these process (or at the very least, know about them)…Perhaps, from the start, embed this project more firmly with what is already ongoing in Kenya (e.g. ensure that objectives are aligned, participate in joint meetings, find ways to provide regular updates and request feedback from local partners, etc.)” (academic researcher 6).

## Recommendations for future TDR projects

Here, we provide practical recommendations for those undertaking TDR projects, based on the learning from our reflections on the strengths and weaknesses of our project, and on the existing literature. Although our recommendations are based on our case study on air pollution in Kenya, many of the underlying principles are anticipated to be applicable in other geographic contexts for various sustainability issues, with careful consideration and adaptation. We structure these recommendations using three common project stages (pre-funding, funded period (planning/doing) and post-funding), though we acknowledge that this is a simplified version of reality (for example some projects will include seed funding). However, structuring the recommendations in this temporal way is intended to enable these recommendations to be more effectively operationalised in future TDR projects. Our recommendations highlight that the foundations for a successful TDR project are made at the pre-funding and planning stages of the project, since the actions taken here underpin the latter stages of the project where the work is actually undertaken.

### Pre-funded period

In preparing for a new TDR project, it is imperative to build a team of academic and non-academic stakeholders ensuring all relevant disciplines and areas are represented (Lang et al. 2012), that there is sufficient local representation (Schmidt and Neuburger 2017), and that equality, diversity and inclusion are taken into consideration (Pischke et al. 2019). Identifying key actors takes time, particularly in contexts such as ours where there were many individuals and groups with low(er) formalisation (e.g. residents of the informal settlement, civil society organisations). Budgets must be appropriate for people’s time, being realistic about how time-consuming TDR is (Thompson et al. 2017; Steger et al. 2021). Following critical reflection of our own project, ensure that beyond the ‘doing’ of the research, there is sufficient time budgeted for team members to participate in reflective processes, undertake the vital knowledge integration stage of TDR and to produce outputs for various academic and non-academic audiences. While some team members may be able to work on outputs beyond the funded project period, many will not have this privilege, impacting on equality and representativeness, and ultimately the overall outcomes of the project. Therefore, provisions should be made at this stage to assist in participation, for example, ensure that output drafting takes place throughout the funded period of the project. Incorporate stakeholders’ views into the funding bid, paying them for their time if possible, to ensure that the project meets their needs. If funders allow, include a flexible budget for co-created activities, as we did with our mini-projects, to allow the project to adapt to stakeholders’ needs. In terms of project timelines, set aside enough time for knowledge integration in later phases of the project (Hoffman et al. 2019; Lang et al. 2012) and prepare non-academic stakeholders for (commonly) slower academic timescales, as we needed to do with our community partners.

### Funded period: planning

Speed up academic bureaucracy as much as possible, e.g. signing of contracts, since our reflection diaries highlighted frustration for some around these aspects. Conduct preparatory work (e.g. stakeholder mapping) to identify power imbalances at different levels (e.g. north–south; intra community; gender-based) and begin planning for how to address these during project implementation. Do not underestimate the importance of building trusting relationships among the team (Stauffacher et al. 2008). Once the project begins, set aside the required time to do this and enable team members to get to know each other (Schmidt and Neuburger 2017), both formally and informally. In our project, activities including sharing food and playing games were used to build relationships and reflection diaries highlighted how important these were for building trust and understanding. Such connections are imperative when projects encounter challenges or when initial enthusiasm and excitement might usually begin to wane. Our reflection diaries also highlighted the importance of selecting appropriate meeting venues within the community, to reduce travel times, increase convenience and ideally enable all team members to better understand the context. A facilitator should be used for meetings and workshops to ensure all voices are heard (Lang et al. 2012). As well as identifying clear roles for all team members (Lang et al. 2012), consider together how people wish to work on the project. We recommend using an ‘interdisciplinary working contract’ (or similar mechanism) to outline how the team wishes to work together collaboratively, and to ensure shared project expectations. We would recommend revisiting the contract as needed throughout the project, e.g. to update it or to use it to help mitigate conflicts. Our reflection diaries highlighted the importance of considering issues of intellectual property and ownership of project ‘outputs’ in advance of the start of the project and reinforcing these along the way. As our reflection diaries highlighted, there is huge potential for capacity building through TDR. This could be explicitly addressed within projects to maximise the benefits. Potential questions to ask team members include What unique skills and experiences do you bring to the project? Which skills would you like to learn through participating in the project? Revisit these questions periodically to highlight progress in learning and where gaps remain; this may help to maintain team members’ enthusiasm for the project. Ensure that the project is designed to deliver both academic and stakeholder impacts and benefits (Lang et al. 2012; Little 2017), and design ways to collate the project’s scientific and societal impacts as the project progresses.

### Funded period: doing

Explore the topic from different angles with the team using various methods, e.g. photovoice, participatory mapping, ethnographic methods (Steger et al. 2021). In our project we particularly found community walks, art and storytelling to be useful methods; reflection diaries highlighted that such methods helped to break down the barriers between stakeholders by moving people out of their comfort zones and usual ways of working. Consider having a ‘journal club’ for sharing articles (academic or otherwise) to build a common understanding of the context and issues. Use reflection diaries (or similar mechanism) for formative evaluation throughout the project, as they allow continuous monitoring and adjustment of project activities. It is important to ensure the diaries are simple for people to complete and submit (e.g. using audio, online or written forms, or a combination of these), and for them to be analysed in near real-time. Budget adequately for everyone’s time to complete the reflection diaries—if we had done this beyond the workshop, this may have helped to increase the response rate from community partners. Keep in touch regularly using appropriate communication mechanisms (Ding et al. 2020). As we found in our project, these may include more informal tools, e.g. WhatsApp, but boundary-setting will be important to maintain work/life balance. Given the (commonly) long funding cycle timescales, identify potential future funding opportunities early to continue collaborative activities (if appropriate).

### Post-funding

Whilst this phase of the project is very important, it is often overlooked in TDR, as was the case in our project. There may be expectations from various team members at this stage, e.g. for project activities to be continued or for writing journal articles; however, we found it was important to have realistic expectations of people’s ability to participate after the project has ended. Understandably, this was particularly the case for our community partners, who were unable to further participate without funding. Many of the societal and scientific impacts of TDR projects will not be realised until after the project has finished, but designing simple data collection tools to document impact(s) in the planning stages of the project makes it easier to collate these throughout (and beyond) the project.

## Conclusion

TDR projects are key for addressing complex global sustainability problems, such as those outlined in the SDGs, as they bring together diverse voices and different ways of knowing to solve problems. However, the use of such approaches can be challenging for a variety of reasons. We used reflective diaries throughout our TDR project about air pollution in an informal settlement in Nairobi, Kenya, to reflect on some of these challenges.

Our reflective diaries enabled us to undertake both a formative evaluation of the project and a summative evaluation of the challenges and opportunities afforded by the TDR approach in this context. Discussing (anonymised) diary entries in team meetings created a space for open discussion and learning about the challenges. By acting on feedback given in reflective diaries in near real-time, we could continuously learn, adapt and improve the project. Diaries were found to be useful by the team, with several using them in subsequent projects. For researchers interested in implementing such an approach in future TDR projects, we recommend developing a contextually appropriate reflective diary collection mechanism (e.g. paper survey, App, online form) at the start of the project, prompting the team to complete the diaries at regular intervals (or specific project time-points), analysing the diaries in as close to real-time as possible to ensure points raised can be acted upon, and collectively discussing anonymised entries to maximise team learning.

The Lang et al. (2012) framework for TDR projects enabled us to reflect critically on the approaches used in our project. Drawing on insights from the reflective diaries, we identified practical mechanisms that supported the work at each of the three key project stages: pre-funding, during the funding, and post-funding, as well as aspects that limited the potential of the project.

A variety of actors have a role to play in putting our recommendations into practice, including those administering and funding projects. In particular, funders need to have a more flexible approach to commissioning and funding work that involves non-academic partners, acknowledging that TDR projects will likely require additional funding to co-develop research plans, and a higher degree of flexibility. Based on our experiences we would recommend funders consider developing specific requirements for TDR projects, for example, ensuring projects budget for knowledge integration and provide plans for applying the knowledge generated. Funders should also insist that sufficient budget is allocated for formative and summative evaluation, so that projects can learn and improve as they go along, as well as share those learnings with future projects. As we have shown, reflective diaries can play an important part in this evaluation, but support is needed to ensure all actors can fully participate in them.

Together, our practical recommendations for undertaking TDR projects can help to support action to address some of the key multidimensional socio-environmental crises that our planet is currently facing, encouraging progress towards the achievement of the SDGs.

## Supplementary Information

Below is the link to the electronic supplementary material.

Supplementary file1 (DOCX 238 KB)

## Data Availability

Data from the reflective diaries are not available.
