# Supplementary figures and images for: From reflection diaries to practical guidance for transdisciplinary research: learnings from a Kenyan air pollution project
Source: Sustain Sci. 2023 Apr 19;18(3):1429–44. doi: 10.1007/s11625-023-01317-0 (PMC10113976; doi:10.1007/s11625-023-01317-0)

Supplementary material


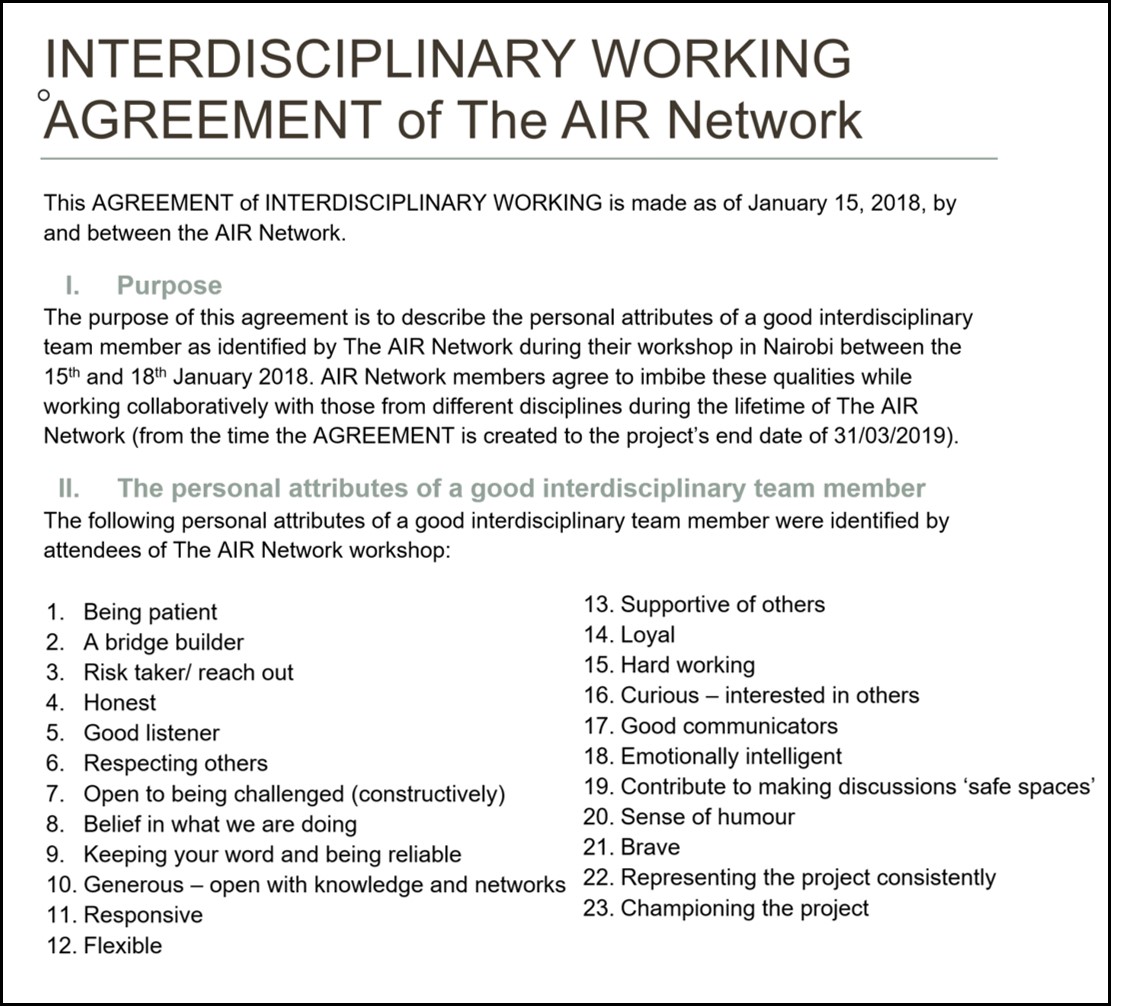

Supplement: Supplementary file 1 — Supplementary file1 (DOCX 238 KB) [file 11625_2023_1317_MOESM1_ESM.docx]
